# Increased IL-1α expression in chronic rhinosinusitis with nasal polyps

**DOI:** 10.1007/s00405-022-07640-z

**Published:** 2022-09-27

**Authors:** Yujie Cao, Xianting Hu, Chun Zhou, Keqing Zhao, Yaoming Zheng, Wenxiu Jiang, Dehui Wang, Huabin Li

**Affiliations:** Allergy Center, Department of Otolaryngology, Affiliated Eye and ENT Hospital, Fudan University, No. 83, Fenyang Road, Shanghai, 200031 China

**Keywords:** Chronic rhinosinusitis, Nasal polyps, Cytokine, IL-1α, Neutrophil

## Abstract

**Purpose:**

To examine whether and how interleukin (IL)-1α is involved in chronic rhinosinusitis with nasal polyps (CRSwNP).

**Methods:**

Nasal polyp (NP) and control tissues were collected from CRSwNP patients and control subjects. The expression of IL-1α and other proinflammatory cytokines (IL-1β, IL-8 and IL-13, etc.), as well as neutrophil and eosinophil accumulation, were examined in sinonasal tissues using immunohistochemical (IHC), immunofluorescent (IF) staining, qPCR, and Luminex, respectively. Moreover, the regulation of IL-1α expression and its effects on other proinflammatory cytokines were evaluated in cultured nasal epithelial cells (NECs).

**Results:**

The mRNA and protein levels of IL-1α were significantly higher in NP tissues compared to that in control tissues. IL-1α in polyp tissues was mainly located in epithelial cells and neutrophils. Polyps IL-1α level was significantly associated with IL-8, IL-1β, IL-6, IL-4 and IL-13 production, as well as tissue neutrophil infiltration. Moreover, poly (I:C), lipopolysaccharides, Flagellin, R848 and cytokines (IL-4, IL-5, and IL-13) significantly increased the expression of IL-1α in cultured NECs in vitro, and recombinant IL-1α significantly promoted production of IL-8 and CXCL1 in cultured NECs.

**Conclusions:**

These findings provided the evidence that IL-1α were significantly increased in NP tissues, which may contribute to tissue neutrophilia in CRSwNP patients in China.

## Introduction

Chronic rhinosinusitis with nasal polyps (CRSwNP) is a heterogeneous chronic inflammatory disorder characterized by visible formation of nasal polyp (NP) with massive tissue edema and distinct inflammatory endotype [1]. In western countries, the majority of CRSwNP patients have previously been reported to be a Th2-typed inflammatory profile with significant tissue eosinophilia, whereas neutrophilic inflammation and mixed Th1/Th17 inflammatory profiles are observed prominently in Asian CRSwNP patients [2]. However, recent evidences show activated neutrophils are significantly elevated in both Asian and Caucasian CRSwNP patients [3–5]. In China, we previously demonstrated that adult CRSwNP patients with enhanced tissue neutrophilia show poor respond to oral corticosteroids [3], and Liao et al. found increased IL-8 expression and more patients with difficult-to-treat CRSwNP in clusters with severe or moderate neutrophilic inflammation by performing a cluster analysis based on clinical variables and pathological features [6]. More recently, Kim et al. reported subepithelial neutrophil infiltration predicts a poor surgical outcome in Korean CRSwNP patients [7]. However, the key factors underlying tissue neutrophilia in these CRSwNP patients are not yet completely understood.

Interleukin (IL)-1 is a family of robust pro-inflammatory cytokines with an exceedingly narrow margin between effectivity and toxicity which needs to be strictly regulated in humans [8]. There are two major members of IL-1, IL-1α and IL-1β. IL‐1α could be produced by epithelial, mesenchymal, and hematopoietic cells, while IL‐1β is primarily produced by monocytes, macrophages, and neutrophils [9]. We previously showed IL-1β was upregulated in neutrophilic CRSwNP following NLRP3 inflammasome activation, which may contributes to NP neutrophilic inflammation [10], and an association between IL1A polymorphism and chronic rhinosinusitis has been demonstrated by other groups as well [11], but the possible role of IL-1α in the pathogenesis of CRSwNP remains unclear. This study thus aimed to investigate the expression of IL-1α and its association with tissue neutrophilia in Chinese CRSwNP patients.

## Materials and methods

### Subjects

This study was approved by the ethical committee of Eye and ENT Hospital, Fudan University, and written informed consent was obtained from all the subjects. All 45 CRSwNP patients were recruited from the Department of Otolaryngology, Eye and ENT hospital, Fudan University. The diagnosis of CRSwNP was made according to the international EPOS consensus [1]. As normal controls, 29 non-atopic patients undergoing septoplasty for nasal septum deviation, endoscopic optic nerve decompression because of traumatic optic neuropathy, or transnasal skull surgery because of anterior skull base tumors were enrolled. The atopic status of the patients and normal controls were evaluated by allergen skin prick tests or serum specific IgE assay. The diagnosis of asthma was made based on case history and pulmonary function test or a prior physician diagnosis. Subjects with acute infection, immunodeficiency, antrochoanal polyps, or allergic fungal sinusitis were excluded from the study.

The demographic characteristics of the subjects are listed in Table 1. NP tissues from CRSwNP patients and sinonasal tissues from normal controls were obtained during surgery. Tissue samples were separated into several portions for RNA and protein extraction, immunohistochemical (IHC) or immunofluorescence (IF) staining. Due to the limit of tissues, not all samples were included in every study protocol.

**Table 1:** Clinical characteristics of subjects

Characteristic	Control	CRSwNP
No. of subjects	29	45
Age (y), median (range)	39 (15–80)	45 (19–72)
Sex (male/female)	21/8	27/18
Atopy, no	0	19
Asthma, no	0	10
Lund–Mackay CT score, mean (SD)	–	16.81 (5.21)
Endoscopic score, mean (SD)	–	4.41 (2.08)

### IHC and IF staining

4-μm sections were cut from paraffin-embedded tissues. For IHC staining, the sections were incubated with primary antibodies against IL-1α (1:100, Abcam, Cambridge, MA, USA), IL-1R1(1:800, Abcam), and neutrophil elastase (NE, 1:1000, Abcam) overnight at 4 °C. Thereafter, each section was incubated and visualized using the Dako REAL EnVision Detection System (Agilent Technologies, Santa Clara, CA, USA). The sections were examined via light microscopy by two investigators who were unaware of the clinical data. The number of NE positive cells was counted in 5 high-power fields (HPFs, 400 ×) with prominent inflammation and averaged. The IHC score of IL-1α was analyzed using the IHC Profiler plugin in ImageJ software. For IF staining, sections were incubated with primary antibodies against IL-1α (1:200, Abcam) and neutrophil elastase (NE, 1:400, Abcam) overnight at 4 °C in two rounds of staining, each using a separate fluorescent tyramide signal amplification system. Images were obtained using a fluorescence microscope (Nikon, Tokyo, Japan).

### Quantitative real-time polymerase chain reaction (RT-qPCR) analysis

The mRNA expression in sinonasal tissues were evaluated using qPCR analysis. Total RNA from sinonasal tissues was extracted using TRIzol (Invitrogen, Carlsbad, CA, USA) and total RNA of cells was extracted using RNAsimple Total RNA Kit (Tiangen, Beijing, China) in accordance with the manufacturer’s instructions. Reverse transcription was performed to synthesized from 1 μg of total RNA using PrimeScript™ RT Master Mix (TaKaRa, Shiga, Japan). mRNA expression was determined using the ABI PRISM 7500 Detection System (Applied Biosystems, Foster City, CA) with SYBR Green Pro Taq HS qPCR Kit (Accurate Biology, Hunan, China). Primers were chosen based on the published data. Target gene expression was normalized to glyceraldehyde 3-phosphate dehydrogenase (GAPDH) and analyzed using the 2^−ΔΔCt^ method. The primer sequences are listed in Table 2.

**Table 2:** Primer sequences for RT-qPCR

Gene	Forward primer (5′–3′)	Reverse primer (5′–3′)
IL-1α	AGATGCCTGAGATACCCAAAACC	CCAAGCACACCCAGTAGTCT
IL-1R1	ATGAAATTGATGTTCGTCCCTGT	ACCACGCAATAGTAATGTCCTG
IL-8	ACTGAGAGTGATTGAGAGTGGAC	AACCCTCTGCACCCAGTTTTC
CXCL1	TCAATCCTGCATCCCCCATAGTTA	GTGAGCTTCCTCCTCCCTTCT
GAPDH	GAGTCAACGGATTTGGTCGT	TTGATTTTGGAGGGATCTCG

### Luminex and enzyme linked immunosorbent assay (ELISA)

Tissues were homogenized and lysed in RIPA buffer supplemented with protease inhibitors purchased from Beyotime Biotechnology. BCA kits were used to detect total protein concentration. The levels of IL-1α, IL-8, and C–X–C motif chemokine 1 (CXCL1) in tissues or cell-free culture supernatants were determined using Luminex kits (R&D Systems, Minneapolis, MN, USA) and ELISA kits (Neobioscience, Shenzhen, China) according to the manufacturer’s protocol, respectively.

### Cell culture and stimulation

Human nasal epithelial cells (HNEpCs) were purchased from PromoCell and grown in minimal essential medium supplemented with 10% fetal bovine serum and Antibiotic–Antimycotic (1:100, Gibco, Grand Island, NY, USA). When a confluence of 80–90% was reached, the cells were washed with PBS, and were treated with Pan3CSK4 (5 μg/mL, Invivogen), poly (I:C) (20 μg/mL, Sigma-Aldrich, St. Louis, MO), Lipopolysaccharides (LPS, 10 μg/mL, Invivogen), Flagellin (200 ng/mL, Invivogen), R848 (5 μg/mL, MedChemExpress, Monmouth Junction, NJ, USA), IFN-γ (50 ng/mL, PeproTech, Cranbury, NJ, USA), IL-4 (50 ng/mL, PeproTech), IL-5(50 ng/mL, PeproTech), IL-6 (50 ng/mL, PeproTech), IL-8 (100 ng/mL, PeproTech), IL-13 (50 ng/mL, PeproTech), indeno(1,2,3-cd)pyrene (IP, 10 nM, Sigma-Aldrich), and multiple concentrations of IL-1α (PeproTech) as noted, respectively. In some experiments, cells were pre-incubated with dexamethasone (DEX, Sigma-Aldrich) for 2 h.

### Statistical analysis

Data were presented as medians and interquartile ranges, or as mean and standard error of the mean. Data were carried out using Prism GraphPad (Prism 8, GraphPad Software, San Diego, CA, USA). The data were analyzed using the Mann–Whitney *U* test or Student’s *t* test. The Pearson’s or Spearman’s correlation coefficient test were used to assess the associations between data sets. A *P* value of less than 0.05 was considered as statistically significant.

## Results

### Expression of IL-1α in NP tissues of CRSwNP patients and sinonasal tissues of normal controls

**Fig. 1:**
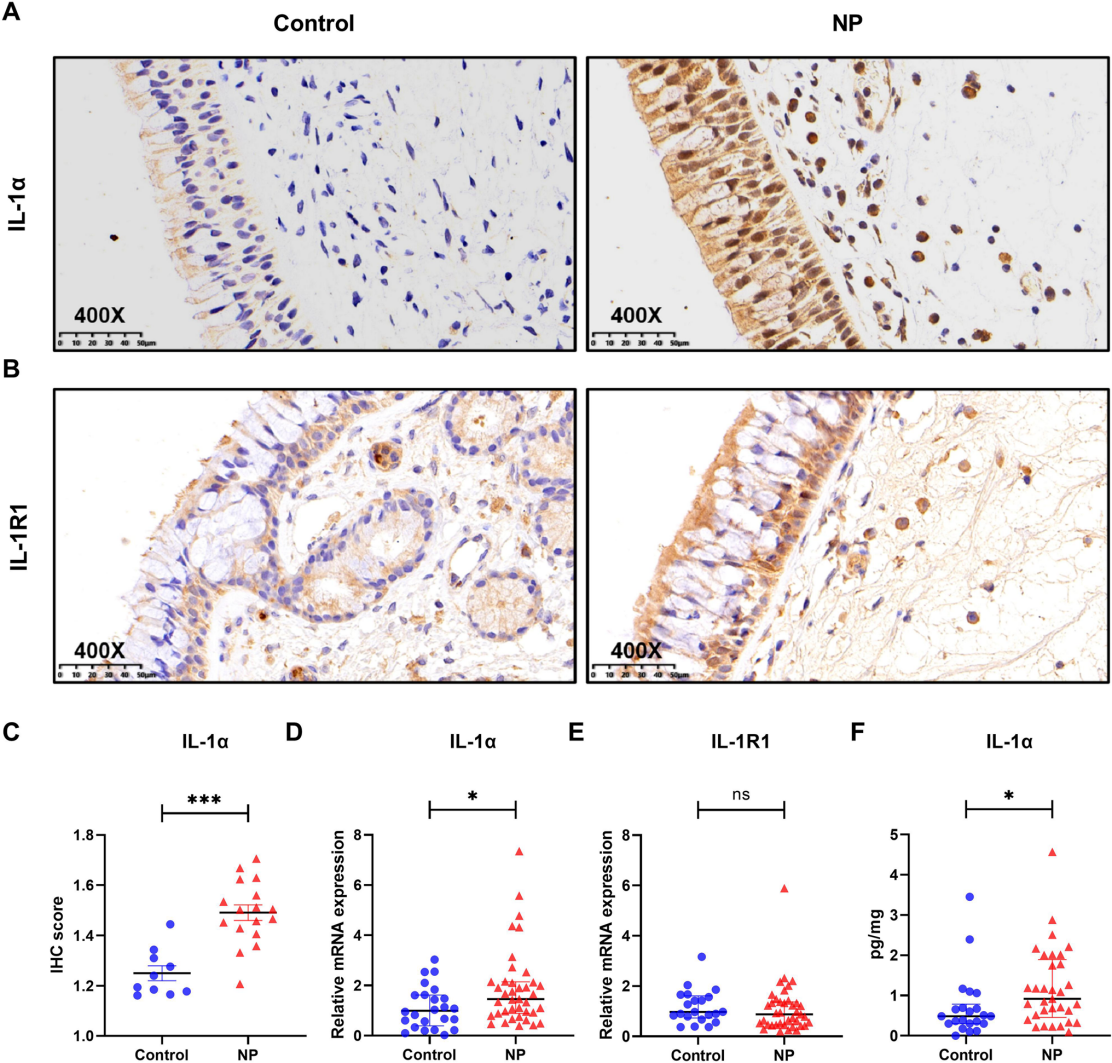
Increased expression of IL-1α in nasal polyp tissues (NP) in CRSwNP patients. **A**, **B** Representative immunohistochemistry (IHC) staining for IL-1α and IL-1R1 in control and polyp tissues. Scale bar, 50 μm. **C** IHC scores of IL-1α staining in control tissues (*n* = 10) and polyp tissues (*n* = 17). **D**, **E** mRNA levels of IL-1α and IL-1R1 in control tissues (*n* = 24 and 21, respectively) and polyp tissues (*n* = 39). **F** Protein level of IL-1α in control tissues (*n* = 22) and polyp tissues (*n* = 33). *P < 0.05, ***P < 0.001

**Fig. 2:**
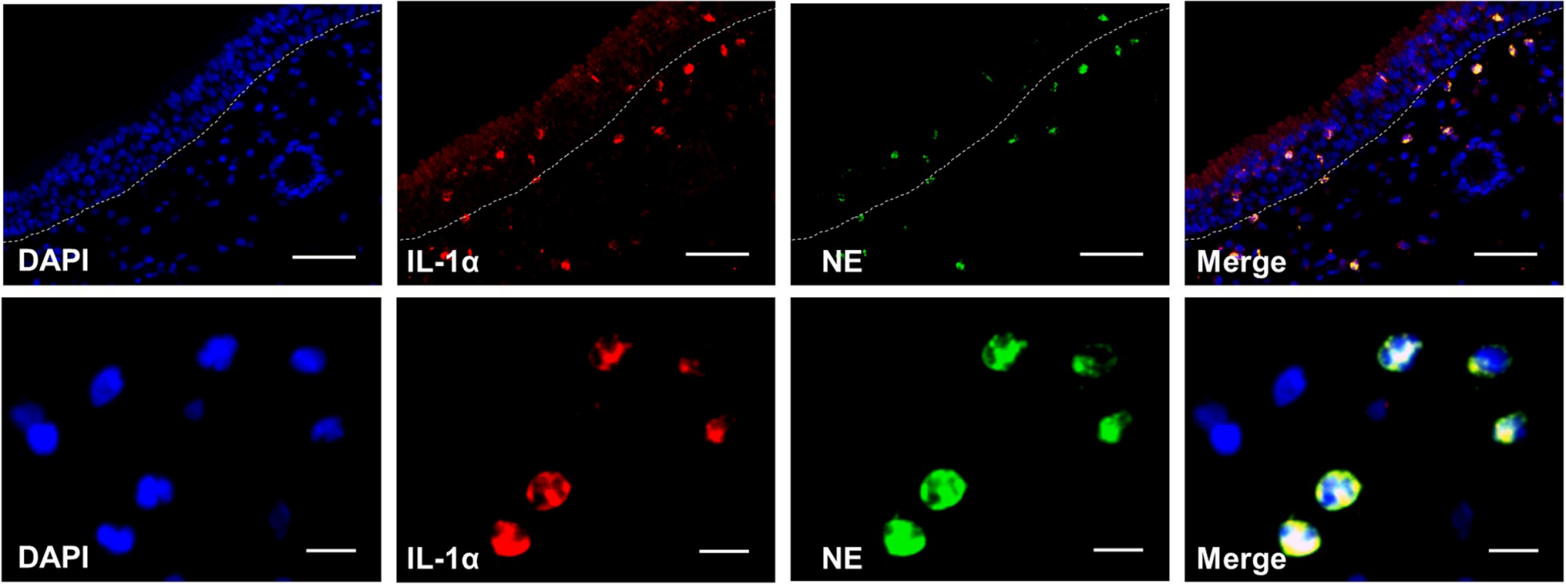
Immunofluorescence staining of IL-1α in epithelial cells and neutrophils of polyp tissues in CRSwNP patients. Representative immunofluorescence staining of IL-1α (red) and neutrophil elastase (NE, green) in polyp tissue. Dotted lines indicate the epithelial cell layer boundary. Scale bars for top images, 50 μm. Scale bars for bottom images, 20 μm

As shown in Fig. 1, IL-1α and its receptor IL-1R1 were mainly localized in nasal epithelium and the stromal cells, as determined by IHC staining. The IHC score of IL-1α in NP tissues was significantly higher than that in control tissues (*P* < 0.01). Be consistent with the IHC results, the mRNA level of IL-1α but not IL-1R1 in NP tissues was significantly increased compared to control tissues (*P* < 0.05). As to the protein level, we found protein level of IL-1α was also significantly increased in NP tissues compared to control tissues (*P* < 0.05). We further examined the cellular sources of IL-1α in NP tissues by IF staining. As shown in Fig. 2, we found both nasal epithelial cells and neutrophils were able to produce IL-1α in NP tissues.

### Association of IL-1α protein level with inflammatory cells and cytokines in sinonasal tissues

**Fig. 3:**
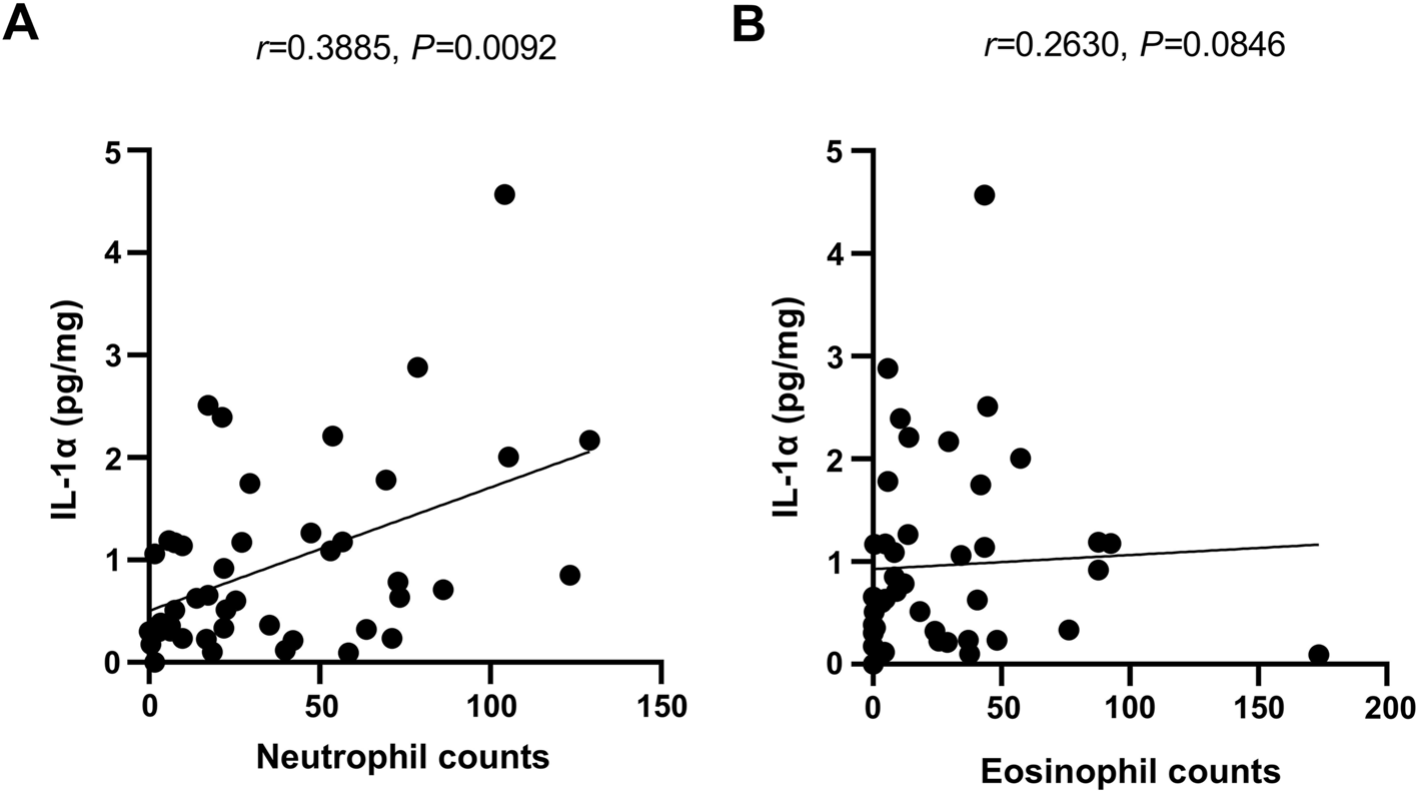
Association of IL-1α level with neutrophil infiltration in sinonasal tissues. The protein level of IL-1α is significantly associated with the number of neutrophils, but not eosinophils, in sinonasal tissues (*n* = 44)

**Fig. 4:**
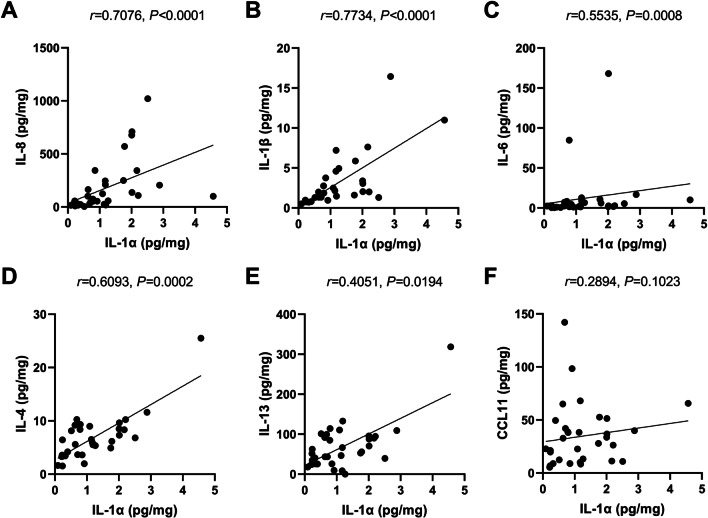
Association of IL-1α protein level with various pro-inflammatory markers in polyp tissues of CRSwNP patients. The protein level of IL-1α is significantly associated with IL-8 (**A**), IL-1β (**B**), IL-6 (**C**), IL-4 (**D**), and IL-13 (**E**), but not CCL11 (**F**), in polyps tissues of CRSwNP patients (*n* = 33)

We then evaluated the association of IL-1α protein level with the infiltrated neutrophils and eosinophils in sinonasal tissues. As shown in Fig. 3, we found IL-1α protein level was significantly associated with neutrophil number, but not eosinophil number, in sinonasal tissues(*P* < 0.05). Moreover, we found the protein level of IL-1α was significantly associated with IL-8, IL-1β, IL-6, IL-4, IL-13 but not CCL11 in NP tissues of CRSwNP patients (*P* < 0.05) (Fig. 4).

### Regulation of IL-1α expression in cultured epithelial cells after stimulation

**Fig. 5:**
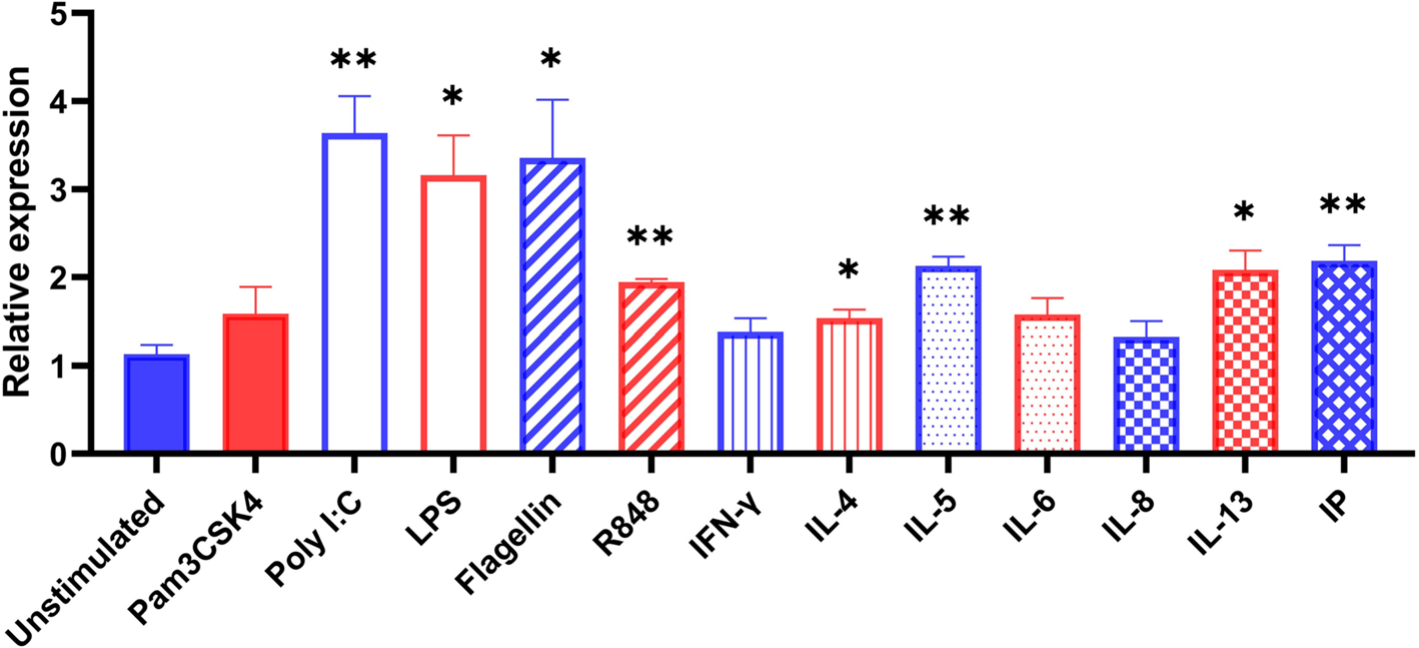
Expression of IL-1α in cultured nasal epithelial cells in response to different stimuli. The mRNA expression of IL-1α in cultured human nasal epithelial cells (HNEpCs) after stimulation. HNEpCs were cultured alone or with TLR agonists (Pan3CSK4, poly (I:C), LPS, Flagellin, R848), cytokines (IFN-γ, IL-4, IL-5, IL-6, IL-8, IL-13) or a major environmental pollutant (IP) for 3 h (*n* = 3). *P < 0.05, **P < 0.01

We next examined the mRNA expression of IL-1α in cultured human nasal epithelial cells (HNEpCs) after stimulation. As shown in Fig. 5, cultured HNEpCs were stimulated with TLR agonists (Pan3CSK4, poly (I:C), LPS, Flagellin, R848), cytokines (IFN-γ, IL-4, IL-5, IL-6, IL-8, IL-13) or a major environmental pollutant (IP) for 3 h and harvested. As to the TLR agonists, poly (I:C), LPS, Flagellin and R848 were able to significantly increased the mRNA expression of IL-1α in cultured HNEpCs (*P* < 0.05). As to the cytokines, we found IL-4, IL-5 and IL-13 were able to significantly increased the mRNA expression of IL-1α in cultured HNEpCs (*P* < 0.05). Moreover, a major environmental pollutant (IP) was shown to significantly increased the mRNA expression of IL-1α in cultured HNEpCs (*P* < 0.05).

### Expression of IL-8 and CXCL1 in cultured epithelial cells after stimulation with recombinant IL-1α

**Fig. 6:**
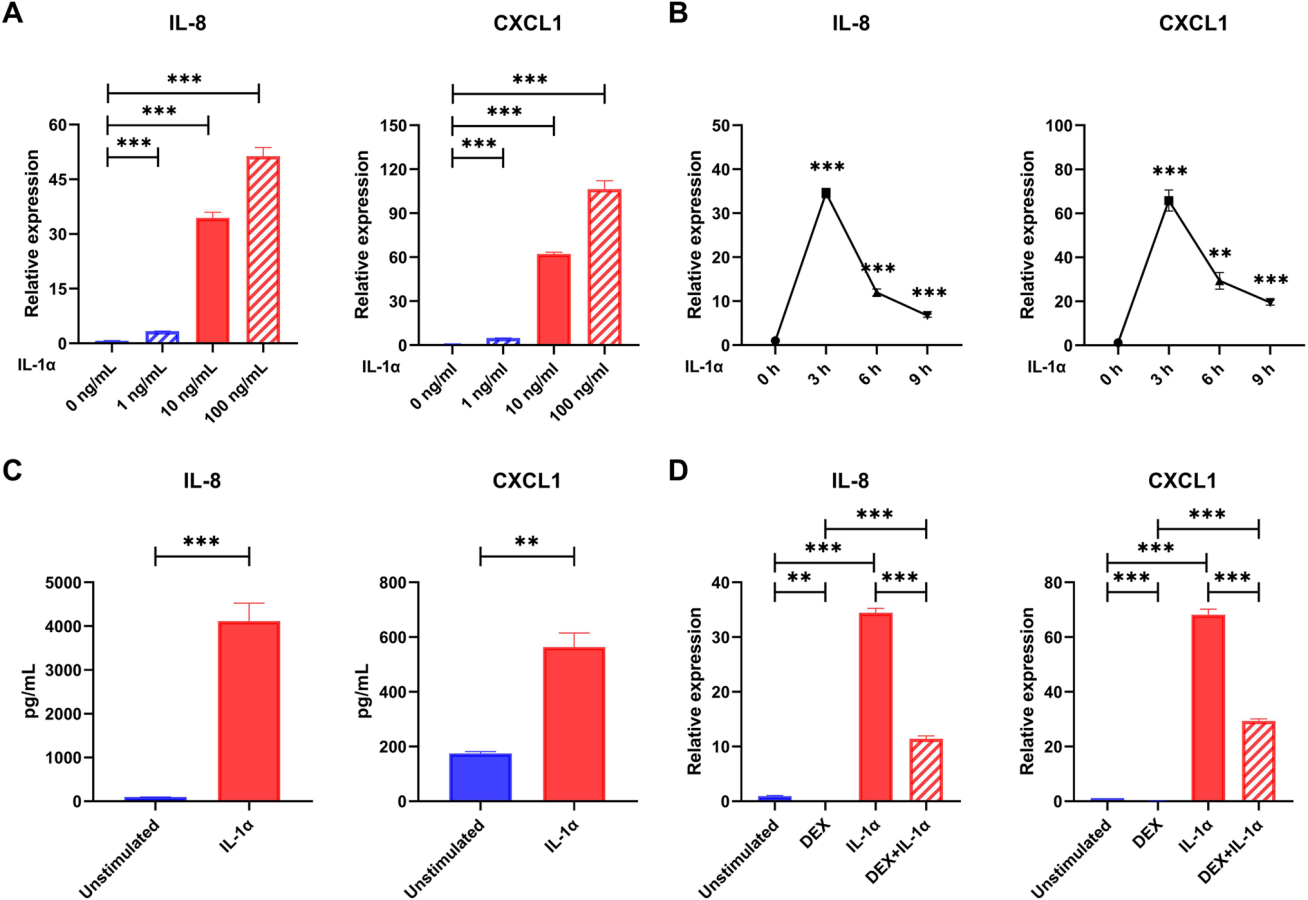
IL-1α induced the production of IL-8 and CXCL1 in cultured nasal epithelial cells. **A** mRNA levels of IL-8 and CXCL1 were significantly increased in cultured human nasal epithelial cells (HNEpCs) after stimulated with different concentrations of IL-1α for 3 h (*n* = 3). **B** mRNA levels of IL-8 and CXCL1 were significantly increased in cultured HNEpCs after stimulated with IL-1α (10 ng/mL) for 3, 6 and 9 h, respectively (*n* = 3). **C** Protein levels of IL-8 and CXCL1 were significantly increased in cell-free culture supernatants of HNEpCs after stimulated with IL-1α (10 ng/mL) for 48 h (*n* = 3). **D** Corticosteroid suppressed the mRNA levels of IL-8 and CXCL1 in cultured HNEpCs in response to IL-1α stimulation. HNEpCs were pre-incubated with DEX (80 ng/mL) for 2 h and then stimulated with IL-1α (10 ng/mL) for 3 h (*n* = 3). **P < 0.01, ***P < 0.001

We finally examined the expression of IL-8 and CXCL1 in cultured nasal epithelial cells after stimulation with recombinant IL-1α. As shown in Fig. 6, we found the mRNA levels of IL-8 and CXCL1 were significantly increased in cultured HNEpCs after stimulated with IL-1α in a time (peak point at 3 h) and dose-dependent manner. Consistently, the protein levels of IL-8 and CXCL1 were significantly increased in cell-free culture supernatants of HNEpCs after stimulated with IL-1α. When corticosteroid was added priorly, we found IL-1α-induced mRNA levels of IL-8 and CXCL1 in cultured HNEpCs were significantly suppressed by DEX (*P* < 0.01).

## Discussion

In this study, we provided the evidence that the mRNA and protein levels of IL-1α were significantly increased in NP tissues of CRSwNP patients, which was positively associated with tissue neutrophil accumulation. These findings might improve our understanding on how IL-1α is involved in the pathogenesis of neutrophilic mucous inflammation in Eastern CRSwNP patients.

CRSwNP is well-known to be characterized by type 2 inflammation and tissue eosinophilia [1], whereas the pathogenesis of neutrophilic CRSwNP has not been fully understood. IL-1α is an important trigger for leukocyte recruitment and inflammation in other airway diseases [8], and epithelial cell-derived IL-1α could activate the expression of intercellular adhesion molecule 1 on endothelial cells in adenovirus type 37-induced airway inflammation [12]. These findings collectively indicate epithelial cell-derived IL-1α is an important trigger for leukocyte recruitment and the exacerbation of inflammation. However, whether IL-1α is involved in the pathogenesis of CRSwNP remains unclear. In this study, we first demonstrated the increased expression of IL-1α in NP tissues was significantly associated with tissue neutrophil accumulation. Interestingly, we found both epithelial cells and neutrophils were the cellular source of IL-1α in NP tissue, indicating a possible self-amplified loop in the neutrophilic inflammation in CRSwNP patients.

We further evaluated the association of IL-1α and other cytokines in NP tissues. As a consequence, we found the protein level of IL-1α was significantly associated with IL-8, IL-1β, IL-6, IL-4, IL-13, but not CCL11 in NP tissues of CRSwNP patients. Previously, we demonstrated that enhanced HIF-1α expression in NP tissues may cause tissue neutrophilia and poor steroid response, and provided an alternative to restore steroid sensitivity by adding clarithromycin [13], and Wang et al. showed that the IL-36γ/IL-36R pathway play a critical role in the development of neutrophilic inflammation and corticosteroid resistance in CRSwNP patients [14]. Collectively, these findings indicate NP neutrophils may be considered a heretofore unrecognized therapeutic target in the management of CRSwNP [15]. Considering both epithelial cells and neutrophils were the cellular source of IL-1α in NP tissue, our findings indicated polyp IL-1α in self-amplified loop may play a central role in the regulation of neutrophilic inflammation of NP tissues.

It has been reported that bronchial epithelial cells are able to release IL-1α to trigger the production of granulocyte macrophage colony-stimulating factor and IL-33 to recruit dendritic cells following TLR4 activation [16]. To further characterize the regulation of IL-1α in polyp epithelial cells in CRSwNP patients, we next examined the expression in cultured NECs in vitro. Consequently, we found IL-1α expression in NECs can be significantly stimulated by both TLR agonists and pro-inflammatory cytokines and environmental pollutant. As to the TLR agonists, poly (I:C), LPS, Flagellin and R848 were able to significantly increased the mRNA expression of IL-1α in cultured HNEpCs, indicating virus, bacteria and other external stimuli were able to stimulate IL-1α production through TLR-mediated pathway. As to the cytokines, we interestingly found Th2 cytokines (IL-4, IL-5 and IL-13) were able to significantly increased the mRNA expression of IL-1α in cultured HNEpCs. These findings may provide a clue that how Th2-dominant eosinophilic inflammation in NP tissues promoted tissue neutrophilia by inducing IL-1α production, to a lesser extent.

In cystic fibrosis, rhinovirus- and hypoxia-induced epithelial necrosis causes IL-1α release and subsequent IL‐8 expression, which may promote neutrophilic inflammation [17, 18]. Since IL-8 and CXCL1 have been known to be the potent chemokines of circulated neutrophil, the underlying mechanism of IL-1α in driving NP tissue neutrophilia, we, therefore, evaluated them in cultured NECs in response to recombinant IL-1α in vitro. As estimated, we found the expression of IL-8 and CXCL1 were significantly increased in cultured HNEpCs after stimulated with IL-1α which can be inhibited by adding corticosteroid. Up to now, only limited studies reported the importance of IL-1α and associated neutrophil in the pathogenesis of CRSwNP. For example, Liu et al. reported increased IL-1α in CRSwNP patients, but they showed activated monocytes as a major source of IL-1α in the NP tissues [19]. In this study, our findings further show both epithelial cells and neutrophils were the cellular source of IL-1α, and established the positive association of IL-1α and neutrophils in NP tissues. Moreover, our results showed both TLR agonists and Th2 cytokines were able to stimulate IL-1α production in cultured epithelial cells, indicating a possible role of epithelial cell derived-IL-1α in linking external stimuli with subsequent IL-8 and CXCL1-mediated neutrophil chemotaxis by a self-amplified loop. These findings thus added a new insight into the possible molecular mechanisms underlying enhanced tissue neutrophilia in response to increased IL-1α in CRSwNP patients, which could be considered as the therapeutic target in the future.

## Conclusions

In the present study, we found increased IL-1α were significantly associated with tissue neutrophil accumulation in CRSwNP patients. Our findings showed both epithelial cells and neutrophils were the cellular source of IL-1α, indicating a self-amplified loop of IL-1α in driving tissue neutrophilia in NP tissues. Moreover, we found both TLR agonists and Th2 cytokines were able to stimulate IL-1α production in cultured epithelial cells, indicating a possible role of IL-1α in linking external stimuli with subsequent IL-8 and CXCL1-mediated neutrophil chemotaxis.
